# Rutin Protects against Pirarubicin-Induced Cardiotoxicity through TGF-*β*1-p38 MAPK Signaling Pathway

**DOI:** 10.1155/2017/1759385

**Published:** 2017-03-06

**Authors:** Yadi Wang, Yang Zhang, Bo Sun, Qing Tong, Liqun Ren

**Affiliations:** ^1^Department of Experimental Pharmacology and Toxicology, School of Pharmacy, Jilin University, 1266 Fujin Road, Changchun, Jilin 130021, China; ^2^The Third Hospital Affiliated to The Jinzhou Medical University, No. 5-2 Heping Road, Jinzhou, Liaoning 120001, China

## Abstract

We investigated the potential protective effect of rutinum (RUT) against pirarubicin- (THP-) induced cardiotoxicity. THP was used to induce toxicity in rat H9c2 cardiomyoblasts. Positive control cells were pretreated with a cardioprotective agent dexrazoxane (DZR) prior to treatment with THP. Some of the cells were preincubated with RUT and a p38 mitogen-activated protein kinase (MAPK) inhibitor, SB203580, both individually and in combination, prior to THP exposure. At a dose range of 30–70 *μ*M, RUT significantly prevented THP-induced reduction in cell viability; the best cardioprotective effect was observed at a dose of 50 *μ*M. Administration of RUT and SB203580, both individually as well as in combination, suppressed the elevation of intracellular ROS, inhibited cell apoptosis, and reversed the THP-induced upregulation of TGF-*β*1, p-p38 MAPK, cleaved Caspase-9, Caspase-7, and Caspase-3. A synergistic effect was observed on coadministration of RUT and SB203580. RUT protected against THP-induced cardiotoxicity by inhibition of ROS generation and suppression of cell apoptosis. The cardioprotective effect of RUT appears to be associated with the modulation of the TGF-*β*1-p38 MAPK signaling pathway.

## 1. Introduction

Anthracyclines, such as pirarubicin (THP), are widely used chemotherapeutic agents in neoplasms such as leukemia, lymphoma, and breast cancer. However, the clinical use of these agents is limited by severe cardiotoxicity [1, 2]. Currently, the iron chelator dexrazoxane (DZR) is the only known drug that alleviates anthracycline-induced myocardial injury, without compromising the antineoplastic efficacy of anthracyclines [3]. However, the carcinogenicity of DZR limits its use [4]. Therefore, novel therapeutic agents with better cardioprotective efficacy and safety are required.

The mechanism of anthracycline-induced myocardial injury is not completely understood. These agents are thought to induce myocardial apoptosis as a result of their interaction with iron, which triggers excessive production of reactive oxygen species (ROS) [5]. Recent studies have demonstrated the effect of anthracyclines on a variety of intracellular signal transduction pathways, which may also contribute to their cardiotoxic effects [6]. Accumulated evidence suggests the involvement of p38 mitogen-activated protein kinase (MAPK) signaling pathway in the regulation of myocardial apoptosis [7–9]. Gu et al. found that doxorubicin (DOX) induced H9C2 cell apoptosis by inhibiting AMPK activation and promoting proapoptotic protein expression through p38 MAPK/p53 signaling [10].

Ghosh et al. [11] reported that DOX, an anthracycline derivative, was shown to activate ROS-dependent p38 MAPK signaling pathway, which led to cardiac apoptosis. Transforming growth factor- (TGF-) *β*1, an upstream mediator of p38 MAPK signal, was shown to activate p38 MAPK via activation of TGF-*β*-activated kinase 1 (TAK1) [12]. Nevertheless, the involvement of TGF-*β*1-p38 MAPK signaling pathway in cardiac apoptosis is still poorly understood.

Rutinum (also known as quercetin-3-O-rutinoside or rutin, RUT) is a dietary flavonoid compound extracted from* Sophora japonica *L. Its immense therapeutic potential can be attributed to its diverse range of properties: clearance of ROS [13], anti-inflammatory action [14], metabolic function improvement [15], neuroprotective effect [13, 16], and antineoplastic properties [17, 18]. However, the potential cardioprotective role of RUT has not been demonstrated.

This in vitro study investigated the effects of RUT against THP-induced cardiotoxicity in rat H9c2 cardiomyoblasts. The role of ROS generation and TGF-*β*1-p38 MAPK signaling pathway in the cardioprotective effect of RUT was assessed.

## 2. Materials and Methods

### 2.1. Reagents

THP was purchased from Shenzhen Main Luck Pharmaceuticals Inc., Guangdong, China. RUT (purity > 98%) was obtained from Nanjing Jingzhu Bio-Technology Co., Ltd., Jiangsu, China. DZR and SB203580 were purchased from Jiangsu Aosaikang Pharmaceutical Co. Ltd., Nanjing, China, and Selleck Chemicals, Houston, USA, respectively. Hoechst 33258 and dichlorodihydrofluorescein diacetate (DCFH-DA) were bought from Nanjing Jiancheng Bioengineering Institute, Jiangsu, China. DMEM-F12 culture medium and fetal bovine serum (FBS) were obtained from GIBCO BRL, USA. Primary antibodies, including anti-p38 MAPK and anti-p-p38 MAPK antibodies, were purchased from ABclonal Technology, Boston, USA. Anti-TGF-*β*1 antibody was purchased from Santa Cruz, CA, USA. Anticleaved Caspase-3, Caspase-7, and Caspase-9 antibodies were purchased from Cell Signaling Technology, Inc., MA, USA.

### 2.2. Cell Culture

Rat H9c2 cardiomyoblasts were obtained from the Cell Bank at the Chinese Academy of Sciences, China, and maintained in DMEM-F12 culture medium supplemented with 10% FBS. Cell cultures were incubated in 5% CO_2_ incubator at 37°C.

### 2.3. Pharmacological Interference

In order to induce cardiomyoblast injury, H9c2 cells were incubated with 5 *μ*M of THP for 24 h. To determine the effect of RUT on cell viability, cells were pretreated with 10, 30, 50, or 70 *μ*M RUT for 1 h, followed by 5 *μ*M of THP incubation for 24 h. In positive control, SB203580 treatment groups, cells were pretreated with 50 *μ*M DZR or 3 *μ*M SB203580 for 1 h, followed by 24 h of THP exposure. In combined treatment group, cells were pretreated with 50 *μ*M RUT and 3 *μ*M SB203580 for 1 h, followed by 24 h of THP exposure.

To understand the mechanism of RUT-mediated cardioprotection, cells were divided into six groups: control, THP, DZR + THP, RUT + THP, SB203580 + THP, and RUT + SB203580 + THP. In THP group, cells were treated with 5 *μ*M THP for 24 h. In DZR + THP, RUT + THP, and SB203580 + THP groups, cells were pretreated for 1 h with 50 *μ*M DZR, 50 *μ*M RUT, and 3 *μ*M SB203580, respectively, followed by 5 *μ*M THP incubation for another 24 h. In RUT + SB203580 + THP group, cells were pretreated for 1 h with 50 *μ*M RUT and 3 *μ*M SB203580, followed by 5 *μ*M THP incubation for another 24 h.

### 2.4. Assessment of Cell Viability

Cell viability was assessed on 3-(4,5-dimethyl-2-thiazolyl)-2,5-diphenyl-2-H-tetrazolium bromide (MTT) assay. Cells were seeded in a 96-well plate. When cells grew to approximately 80% confluence, drugs were administered. Each experimental group was repeated in four wells. After incubation, 100 *μ*L of MTT solution (0.5 mg/mL) was add to the culture medium of each well. Four hours after MTT treatment, culture medium containing MTT solution was removed; the cells were treated with 150 *μ*L of DMSO and placed onto a shaker for 10 min to resuspend the MTT metabolic product. The absorbance was measured at 490 nm by using a microplate spectrophotometer (AOE Instruments V-1900 (Shanghai) Co., Ltd., China). Cell viability was calculated using the following equation:

(1) $$\begin{array}{c} \text{Cell viability}\left(\;\%\;\right)=\left(\;\frac{{\text{Optical Density}}_{Sample}}{{\text{Optical Density}}_{Control}}\;\right)\times 100\%. \end{array}$$

The average cell viability from three independent experiments was recorded.

### 2.5. Determination of Intracellular ROS Concentration

To determine the intracellular ROS level, cells were seeded onto glass coverslips. Drugs were administered when cells grew to approximately 80% confluence. Experiments were performed in a triplicate in each group. Following drug incubation, cells were rinsed twice in phosphate buffer solution (PBS) and further incubated with 10 *μ*M dichlorofluorescin diacetate (DCFH-DA) for 30 min at 37°C. After washing, 5 nonoverlapping areas were randomly selected and micrographs captured under fluorescent microscope (BX50-FLA, Olympus, Japan). The average fluorescence intensity was calculated from five images using ImageJ 1.410 software.

### 2.6. Hoechst Staining

The morphological alterations in apoptotic cells were examined by Hoechst 33258 staining. Briefly, after treatment, cells were washed with PBS and fixed in 4% paraformaldehyde (PFA) for 10 min. After PBS washing, cells were stained with 5 mg/L Hoechst 33258 for 30 min at room temperature and examined under fluorescent microscope (BX50-FLA, Olympus, Japan). Cells with evenly distributed chromatin and uniform blue colored nuclei were considered healthy, while those with condensed (bright blue) or fragmented nuclei were identified as apoptotic cells.

### 2.7. Flow Cytometric Analysis

Cell apoptosis was also determined on Annexin V-FITC/Propidium iodide (PI) staining followed by flow cytometric analysis. H9c2 cells at log phase were collected, prepared as a single-cell suspension, and seeded onto a six-well plate at a density of 1 × 10^5^ cells/well. After drug treatment, cells were collected by centrifugation at 1000 rpm for 5 min. The cells were then washed with PBS, and approximately 1 × 10^5^–5 × 10^5^ cells were suspended in 500 *μ*L of Annexin V binding buffer. Subsequently, 5 *μ*L of Annexin V-FITC solution and 5 *μ*L of PI solution were added to the cell suspension, mixed and incubated for 10 min at room temperature in the dark. The percentage of early cell apoptosis (Annexin V+/PI−) was assessed on flow cytometry (BD Biosciences FACSCalibur, USA).

### 2.8. Western Blot

Cells were seeded onto 60 mm dish. After drug treatment, cells were washed twice with PBS and then lysed with the cell lysis buffer at 4°C for 30 min. Samples were centrifuged at 12,000 rpm for 10 min, and the supernatant was collected for Western blot analysis. Protein concentration was determined on butyleyanoacrylate assay. After SDS-polyacrylamide gel electrophoresis (SDS-PAGE), protein samples were transferred onto a polyvinylidene fluoride (PVDF) membrane, blocked in 5% nonfat dry milk for 60 min, and incubated overnight with anti-TGF-*β*1 (1 : 500), anti-p38 (1 : 1000), anti-p-p38 (1 : 1000), anticleaved Caspase-3 (1 : 1000), Caspase-7 (1 : 1000), or Caspase-9 (1 : 1000) antibodies at 4°C. After 3 washes with Tris buffered saline plus Tween 20 (TBST), samples were probed with secondary antibodies, and immunoblots were visualized on electrogenerated chemiluminescence (ECL) assay. The bands were scanned and the densitometric values of the bands of interest were analyzed by the gel imaging system. Glyceraldehyde 3-phosphate dehydrogenase (GAPDH) was used as internal control.

### 2.9. Statistical Analysis

Data was analyzed using SPSS software version 17.0, and expressed as mean ± standard deviation (SD). Between-group differences were assessed using one way Analysis of Variance (ANOVA). Comparison between the means was performed by LSD* t*-test. A value of *P* < 0.05 was considered statistically significant.

## 3. Results

**Figure 1:**
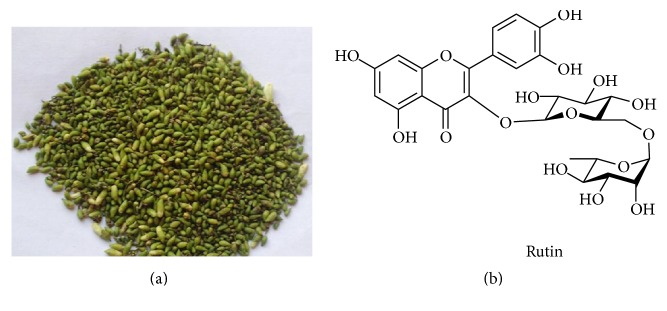
Description of* Sophora japonica* L. and RUT. (a) Gross morphology of* Sophora japonica L. *(b) Chemical structure of RUT. RUT, rutinum.

The gross morphology of* Sophora japonica* L. is presented in Figure 1(a), and the chemical structure of RUT (C_27_H_30_O_16_, molecular weight, 610.52 D), extracted from* Sophora japonica* L., is illustrated in Figure 1(b). We examined the potential effects of RUT in preventing cardiomyoblast injury. THP was used to induce cardiotoxicity in rat H9c2 cardiomyoblasts.

**Figure 2:**
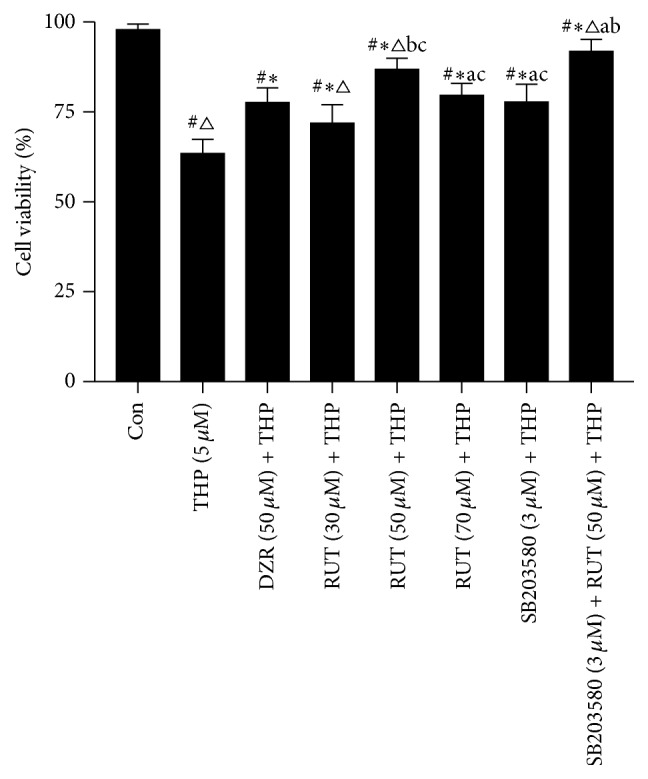
Posttreatment cell viability assessment on MTT assay. ^#^*P* < 0.05 versus control; ^*∗*^*P* < 0.05 versus THP; ^△^*P* < 0.05 versus DZR + THP. ^a^*P* < 0.05 versus RUT (50 *μ*M) + THP; ^b^*P* < 0.05 versus SB203580 + THP; ^c^*P* < 0.05 versus RUT + SB203580 + THP. DZR, dexrazoxane; MTT, 3-(4,5-dimethylthiazol-2-yl)-2,5-diphenyltetrazolium bromide; THP, pirarubicin.

Administration of THP significantly reduced the cell viability as compared to the control (control, 100%; THP, 63.45%  ± 3.94%; *P* < 0.05) (Figure 2(a)). Pretreatment with DZR, a cardioprotective agent, attenuated the decline in cell viability induced by THP, as compared to treatment with THP alone (*P* < 0.05). Moreover, administration of RUT, at the concentrations ranging from 30 to 70 *μ*M, reversed the reduction of cell viability in THP-treated cells (*P* < 0.05).

The most evident cardioprotective effect was observed in cells that were pretreated with 50 *μ*M of RUT (50 *μ*M RUT + THP, 87.83%  ± 4.84%; DZR + THP, 77.61%  ± 4.08%; *P* < 0.05); therefore, this dosage was used in further experiments. Moreover, pretreatment with a specific inhibitor of p38 MAPK, SB203580, also reversed the loss of cell viability induced by THP (*P* < 0.05). Combined administration of RUT with SB203580 produced a synergistic effect in preventing THP-induced cardiotoxicity (*P* < 0.05 versus RUT 50 *μ*M + THP or SB203580 + THP).

**Figure 3:**
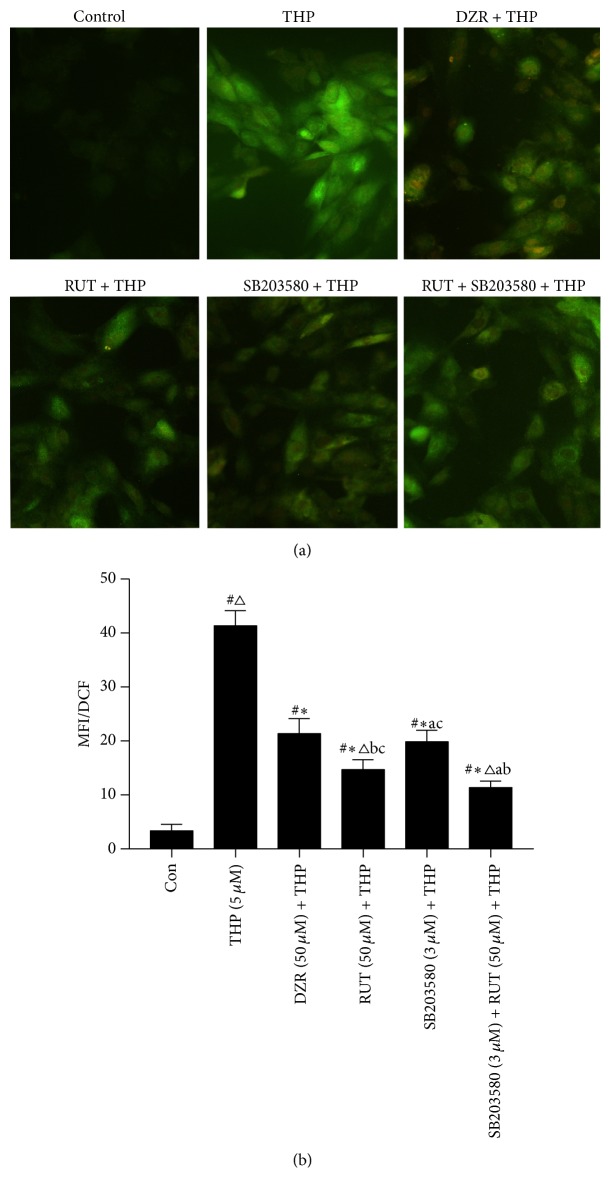
Intracellular ROS concentration. After drug treatment, intracellular ROS concentration was assessed on DCFH-DA probing. (a) Representative images of DCFH-DA staining (original magnification ×200); (b) the average fluorescence intensity was quantified. ^#^*P* < 0.05 versus control; ^*∗*^*P* < 0.05 versus THP; ^△^*P* < 0.05 versus DZR + THP; ^a^*P* < 0.05 versus RUT (50 *μ*M) + THP; ^b^*P* < 0.05 versus SB203580 + THP; ^c^*P* < 0.05 versus RUT + SB203580 + THP. ROS, reactive oxygen species; DCFH-DA, dichlorodihydrofluorescein diacetate; THP, pirarubicin; DZR, dexrazoxane; RUT, rutinum; MFI, mean fluorescent intensity; DCF, 2′,7′-dichlorofluorescein.

The intracellular ROS level was low in control H9c2 cells (Figure 3). After exposure to THP, the intracellular ROS concentration was dramatically elevated as compared to the control (*P* < 0.05). Pretreatment with positive control agent, DZR, significantly decreased THP-mediated ROS generation in cells (*P* < 0.05 versus THP). Compared with DZR, RUT was more potent in reducing ROS production induced by THP (*P* < 0.05 versus DZR + THP). In addition, inhibition of p38 MAPK using SB203580 also yielded a similar effect on ROS level as DZR. Combined administration of RUT with SB203580 appeared to have a synergistic effect in reducing intracellular ROS level.

**Figure 4:**
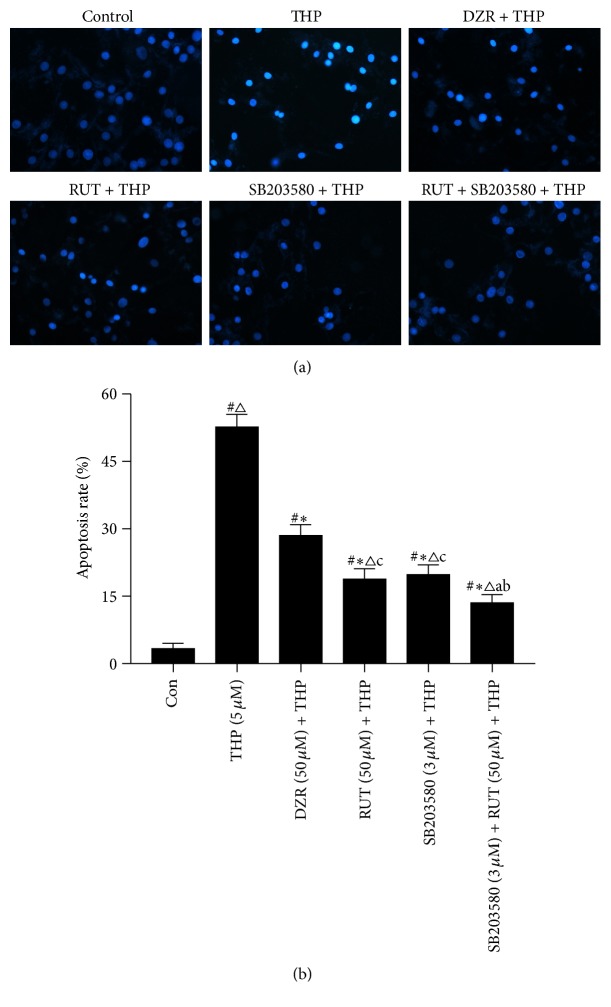
Determination of cell apoptosis by Hoechst 33258 staining. (a) Representative images of Hoechst 33258 staining (original magnification ×200); (b) average percentage of apoptotic cells. ^#^*P* < 0.05 versus control; ^*∗*^*P* < 0.05 versus THP; ^△^*P* < 0.05 versus DZR + THP; ^a^*P* < 0.05 versus RUT (50 *μ*M) + THP; ^b^*P* < 0.05 versus SB203580 + THP; ^c^*P* < 0.05 versus RUT + SB203580 + THP. THP, pirarubicin; DZR, dexrazoxane; RUT, rutinum.

To investigate the potential influence of RUT and SB203580 on cell apoptosis, Hoechst 33258 staining was carried out. THP exposure remarkably increased the proportion of apoptotic cells, as evident from the presence of condensed or fragmented nuclei (*P* < 0.05 versus control) (Figure 4). Administration of DZR significantly suppressed THP-induced cell apoptosis (*P* < 0.05 versus THP). Moreover, RUT and SB203580, either alone or in combination, seemed to be more efficient at inhibiting THP-induced cell apoptosis as compared to DZR (*P* < 0.05 versus DZR + THP).

**Figure 5:**
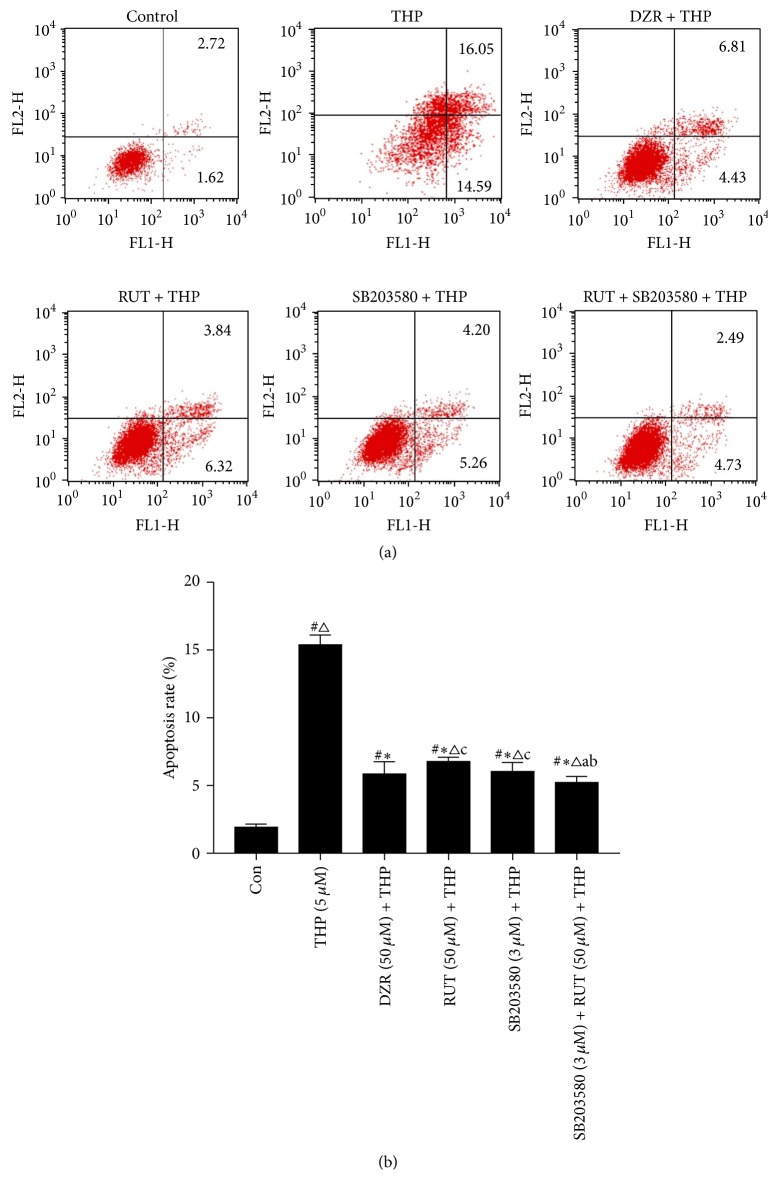
Determination of cell apoptosis on Annexin V/PI staining followed by flow cytometric analysis. (a) Representative data of flow cytometric analysis; (b) average percentage of early apoptotic cells (Annexin V+/PI−). ^#^*P* < 0.05 versus control; ^*∗*^*P* < 0.05 versus THP; ^△^*P* < 0.05 versus DZR + THP; ^a^*P* < 0.05 versus RUT (50 *μ*M) + THP; ^b^*P* < 0.05 versus SB203580 + THP; ^c^*P* < 0.05 versus RUT + SB203580 + THP.

Cell apoptosis was further evaluated by Annexin V-FITC/PI double staining followed by flow cytometric analysis. Similar to the Hoechst staining results, pretreatment with RUT and SB203580, either alone, or in combination, caused a greater reduction in the percentage of apoptotic cells as compared to that by positive control drug DZR (*P* < 0.05 versus DZR + THP) (Figure 5).

**Figure 6:**
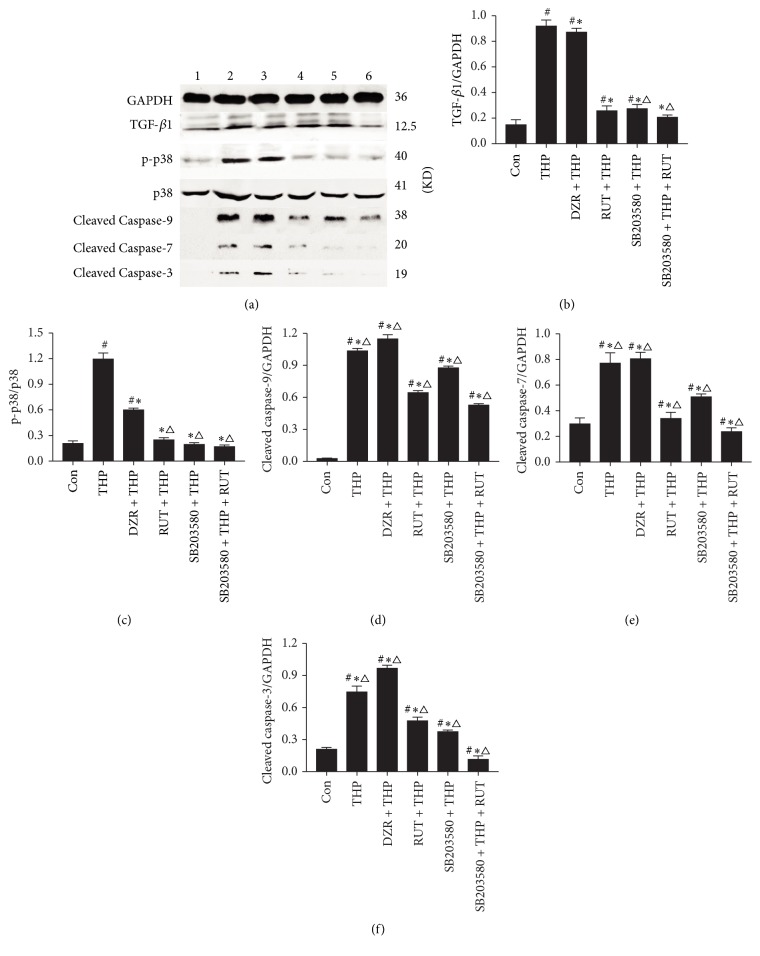
Western blot analysis of protein expression. (a) Representative data of Western blot analysis. Glyceraldehyde 3-phosphate dehydrogenase (GAPDH) was used as internal control. Semiquantitative analysis of TGF-*β*1, p-p38 MAPK, cleaved Caspase-9, Caspase-7, and Caspase-3 levels were presented in (b–f). The relative expression of p-p38 MAPK was normalized to that of total p38 MAPK and others were normalized to that of GAPDH. ^#^*P* < 0.05 versus control; ^*∗*^*P* < 0.05 versus THP; ^△^*P* < 0.05 versus DZR + THP. 1 = control, 2 = THP group, 3 = DZR + THP group, 4 = RUT + THP group, 5 = SB203580 + THP group, and 6 = SB203580 + THP + RUT group. DZR, dexrazoxane; MAPK, mitogen-activated protein kinase; RUT, rutinum; THP, pirarubicin; TGF-*β*1, transforming growth factor-*β*1.

To identify the molecular mechanisms involved in RUT-mediated cardioprotective action, we assessed the protein expression of several crucial regulators of the TGF-*β*1-p38 MAPK signaling pathway. H9c2 cells were incubated with or without 3 *μ*M SB203580 for 1 h and were then exposed to 5 *μ*M THP in the presence or absence of 50 *μ*M RUT. The p38 activation was detected by Western blot analysis using an anti-p-p38 antibody. When cells were exposed to THP, p38 phosphorylation was increased compared with the control (*P* < 0.05 versus control), whereas p38 phosphorylation was significantly decreased by RUT treatment compared with THP (*P* < 0.05; Figure 6). Treatment with DZR reversed the upregulation of the above proteins (*P* < 0.05 versus THP). Additionally, pretreatment with RUT and SB203580, either alone or in combination, efficiently suppressed the THP-induced elevation of the TGF-*β*1, cleaved Caspase-3, Caspase-7, and Caspase-9 proteins (*P* < 0.05 versus THP and DZR + THP). These results suggest that RUT prevents cell apoptosis and protects H9c2 cells through the MAPK/p38 pathway.

## 4. Discussion

RUT has multiple biological and pharmacological properties [19]; however, its potential role in cardioprotection is yet to be clarified. In the present study, RUT was comparatively more effective than DZR in preventing THP-induced toxicity in rat H9c2 cardiomyoblasts. Additionally, the protective effect of RUT appeared to be associated with its ability to scavenge intracellular ROS and inhibit cell apoptosis by modulating the TGF-*β*1-p38 MAPK signaling pathway.

First, we determined the impact of RUT on THP-induced H9c2 cell damage. MTT results revealed that RUT at a dose range of 30 to 70 *μ*M reversed the THP-induced loss of cell viability; the most evident effect was observed at the dose of 50 *μ*M. In accordance with our findings, Zhou et al. [20] demonstrated that pretreatment of RUT greatly alleviated the loss of cell viability in human lens epithelial (HLE) cells exposed to hydrogen peroxide. Others have also reported an antiproliferative effect of RUT, at concentrations between 1 and 100 *μ*M on cultured vascular smooth muscle cells (VSMCs) [21]. This discrepancy in results may be attributed to differences in cell cultures and experimental paradigms used.

Cardiotoxicity is one of the most severe adverse effects of anthracyclines as chemotherapeutic agents. The effect is commonly accompanied by excessive production of ROS [22]. Anthracyclines, such as THP, a derivative of DOX, control iron metabolism, disrupt redox cycling, and result in ROS generation and oxidative stress which is harmful to the heart [22, 23]. RUT is a dietary antioxidant [24]. Here, we found that pretreatment of RUT at a concentration of 50 *μ*M dramatically inhibited the THP-induced elevation in intracellular ROS levels. These results suggest that the cardioprotective action of RUT is likely to be associated with its ability to eliminate ROS. Persistent generation of intracellular ROS may lead to the oxidative stress and induce mitochondrial-associated cell apoptosis in cardiomyocytes [25].

In this study, pretreatment with RUT greatly prevented cell apoptosis of H9c2 cells exposed to THP. Similar antiapoptotic effects have been observed using other plant-derived compounds such as paeoniflorin, which prevented DOX-induced ROS generation and suppressed apoptosis of H9c2 cells [26]. Similarly, another antioxidant, edaravone, was shown to attenuate ROS production, reduce oxidative stress, and inhibit cell apoptosis in H9c2 cells treated with high glucose levels [27].

MAPK signaling cascades, mainly composed of p38 MAPK, c-Jun N-terminal kinase (JNK), and extracellular signal-regulated kinase (ERK), play several functional roles in cardiovascular health and disease [28]. Abnormal activation of MAPK signaling pathway has been observed under different pathological conditions. The three components of MAPK signaling cascades vary in their ability to regulate cardiac myocyte apoptosis. The p38 MAPK and JNK have proapoptotic effect whereas ERK has an antiapoptotic effect [29]. The generation of ROS, accompanied by the activation of p38 MAPK, contributes to apoptosis of H9c2 cells [30]. In this present study, a dramatic upregulation of p-p38 MAPK and its upstream mediator TGF-*β*1 was detected in cells treated with THP, which suggests that activation of TGF-*β*1-p38 MAPK signaling pathway may have contributed to cardiomyocyte apoptosis. Pretreatment with a specific inhibitor of p38 MAPK pathway, SB203580, RUT, or the positive control agent DZR, efficiently inhibited the elevated expressions of TGF-*β*1 and p-p38 MAPK. It seems that RUT itself was sufficient in blocking p38 MAPK pathway. However, administration of RUT and the pathway inhibitor, SB203580, showed a synergistic effect.

RUT is a multifunctional natural product with multiple pharmacological properties [19]. Thus, it is possible that RUT may have multiple intracellular targets in addition to TGF-*β*1-p38 MAPK inhibition. Consistent with our findings, Park et al. [31] reported that RUT prevented ROS production and maintained action potential at the mitochondrial membrane and apoptosis in human dopaminergic SH-SY5Y cells by inhibition of p38 MAPK signaling pathway. In an in vivo study, intraperitoneal injection of RUT attenuated the cyclophosphamide-induced oxidative stress and hepatotoxicity by inhibiting p38 MAPK activation [32].

In conclusion, pretreatment with RUT prevented THP-induced ROS generation and cell apoptosis in cultured H9c2 cells. The effect was mediated via inhibition of the TGF-*β*1-p38 MAPK signaling pathway. Our findings provide basic evidence to understand the cardioprotective effects of RUT. Nevertheless, our study has several limitations. The cause-and-effect relationship between ROS production and cell apoptosis remains unclear. Secondly, although RUT and THP altered the expression of TGF-*β*1-p38 MAPK pathway related proteins, it is not clear whether TGF-*β*1 is an upstream mediator of p38 MAPK. Future studies need to include a TGF-*β*1 receptor antagonist to explore the potential role of TGF-*β*1 blockade on THP-induced cardiotoxicity. Thirdly, the cardioprotective effect of RUT needs to be established in a rodent model of cardiotoxicity.

## Abbreviations

BCA: Butyleyanoacrylate
DCFH-DA: Dichlorodihydrofluorescein diacetate
DMSO: Dimethyl sulfoxide
DOX: Doxorubicin
DZR: Dexrazoxane
FBS: Fetal bovine serum
HLE: Human lens epithelial
MAPK: Mitogen-activated protein kinase
MTT: 3-(4,5-Dimethyl-2-thiazolyl)-2,5-diphenyl-2-H-tetrazolium bromide
PVDF: Polyvinylidene fluoride
PI: Propidium iodide
ROS: Reactive oxygen species
RUT: Rutinum
SDS-PAGE: SDS-polyacrylamide gel electrophoresis
TAK1: Transforming growth factor beta-activated kinase 1
TGF-*β*1: Transforming growth factor-*β*1
THP: Pirarubicin
p-p38: Phosphorylated p38
VSMCs: Vascular smooth muscle cells.
